# Assessment of combined exposure to intermediate-frequency electromagnetic fields and pulsed electromagnetic fields among library workers in Japan

**DOI:** 10.3389/fpubh.2022.870784

**Published:** 2022-07-28

**Authors:** Sachiko Yamaguchi-Sekino, Masao Taki, Miwa Ikuyo, Kaoru Esaki, Atsuko Aimoto, Kanako Wake, Noriko Kojimahara

**Affiliations:** ^1^Work Environment Research Group, National Institute of Occupational Safety and Health, Kawasaki, Japan; ^2^Department of Systems Design, Tokyo Metropolitan University, Hachioji, Japan; ^3^Electromagnetic Compatibility Laboratory, National Institute of Information and Communications Technology, Koganei, Japan; ^4^Strategic Planning Office, National Institute of Information and Communications Technology, Koganei, Japan; ^5^Department of Epidemiology, Shizuoka Graduate University of Public Health, Shizuoka, Japan

**Keywords:** EAS, IF-EMF, library workers, occupational exposure, pulsed EMF

## Abstract

**Objective:**

To assess exposure levels to electromagnetic fields (EMFs) among library workers in Japan, focusing on co-exposure to intermediate-frequency EMF (IF-EMF) and pulsed EMF, to propose a new epidemiological research methodology.

**Methods:**

The evaluated exposure sources were an electromagnetic type-electronic article surveillance gate (EM-EAS, IF-EMF (operating frequency 220 Hz-14 kHz)) and an activator/deactivator of anti-theft tags termed as “book check unit” (BCU, pulsed EMF). Short-term exposures were: (E1) whole-body exposure from the EAS gate when sitting within 3 m; (E2) local exposure to transient IF-EMF while passing through or beside the EAS gate; and (E3) local exposure to a pulsed magnetic field on BCU use. E1–E3 were evaluated based on exposure levels relative to magnetic flux density at the occupational reference level (RL; E1) or as per occupational basic restrictions (BR; E2 and E3) delineated by the International Commission on Non-Ionizing Radiation Protection (ICNIRP) 2010 guidelines. Exposure indices based on mid-term exposure (D1–D3), assuming exposure according to employment on a weekly basis, were used to assess exposure in actual working conditions. D1 represents continuous exposure from an EAS gate when sitting within 3 m of the gate. D2 and D3 represent repeated transient exposures occurring during gate pass or on the operation of a BCU. A link to a web-based questionnaire was distributed to librarians working at all libraries where the authors had mailed institutional questionnaires (4,073 libraries). Four exposure patterns were defined according to various exposure scenarios.

**Results:**

We obtained information on exposure parameters and working conditions from the 548 completed questionnaires. The ICNIRP guideline levels were not exceeded in any of the E1–E3 scenarios. Median of the D1 (% ICNIRP RL × hour/week) was 1, and >85% respondents had values <10. However, the maximum value was 513. Altogether, these results indicate that continuous exposure was low in most cases. The same tendency was observed regarding repeated transient exposure from EM-EAS gates (i.e., the median value for D2 (% ICNIRP BR × gate pass) was 5). However, there were several cases in which D1 and D2 values were >10 times the median. The median of D3 (% ICNIRP BR × BCU operation) was 10, and most respondents' D3 values were greater than their D2 values, although the derived results depended on the assumptions made for the estimation.

**Conclusion:**

We conducted an assessment of combined exposures to IF-EMF and pulsed EMF among library workers in Japan by evaluating both short-term exposures (E1–E3) and exposure indices based on mid-term exposures (D1–D3) assuming actual working conditions per questionnaire results. These results provide useful information for future epidemiological studies.

## Introduction

Intermediate frequency electromagnetic fields (IF-EMF) are electromagnetic fields with frequencies ranging from 300 Hz to 100 kHz (1). Although several studies have examined exposures to IF-EMF from home appliances (2, 3), induction heating cooktops (4, 5), and electronic article surveillance (EAS) systems (6–11), this frequency range has not been comprehensively assessed for safety (12). Few studies have involved IF-EMF safety assessments. In particular, epidemiological studies are largely awaited in this research area (13, 14).

EAS systems are typically installed as anti-theft devices in retail shops and libraries. The gate-type detector (i.e., an EAS gate) is the most commonly used EAS system. EAS systems consist of a detector (i.e., antennas), tags, and an electromagnetic activation and deactivation system. A monitoring zone is created by sending a signal from the transmitter to the receiver at a particular frequency. EAS gates are classified into electromagnetic (EM), acousto-magnetic, self-alarming, radio frequency-operating (RF) types, as well as other systems based on different sensor characteristics and operating frequency ranges. Systems that do not use electromagnetic fields, such as flapper gates, are also commonly installed.

EM-EAS gates are known to be among the strongest sources of sinusoidal continuous wave (CW) IF-EMFs (15). In Japan, many libraries are equipped with this type of EAS gate. Moreover, library workers often handle activators/deactivators for anti-theft tags that use magnetizers/demagnetizers (termed “book check units” (BCUs) in the present study). Previous studies have reported on power frequency EMF exposure generated from BCUs (9, 10, 16). In addition, the waveform of the magnetic field derived from lower frequency demagnetizers was previously reported as a burst-modulated power-frequency field when a label is detected (16). However, as stated in these previous studies, short pulses (pulsed EMFs) were detected in BCU processing and it was previously reported that exposures were likely localized to the hands (16, 17).

In sum, library workers in Japan are exposed to a combination of CW IF-EMF and pulsed-EMF containing IF components. Thus, far, no additional reports have been published regarding exposure to pulsed EMF on the use of BCUs. Hence, the present study assessed exposure to pulsed magnetic fields among library workers in order to fill this knowledge gap.

Our group has previously reported research findings regarding the measurement and exposure assessment of intermediate frequency magnetic fields originating from EAS systems in libraries (18). More specifically, this previous study investigated two types of exposures: transient exposures due to passing through or beside EAS gates, and continuous exposures in the room. However, neither the exposure evaluations for BCUs nor the assessments of exposure in actual working conditions have been implemented as of yet. By addressing this gap in the literature, more accurate exposure assessments that can be used for conducting rigorous, gold-standard epidemiological studies will be generated.

The present study evaluated exposure levels to EMFs among library workers in Japan, focusing especially on co-exposure to IF-EMF (from EAS gates) and pulsed EMF (from BCUs). In doing so, we aimed to propose new methodology for use in epidemiological research on EMFs. For exposure assessment, the results of previous studies were used to generate exposure estimates in combination with answers to the questionnaire survey that was administered in the current study. EAS exposures were also partially estimated using the results of the abovementioned previous study by our group (18). The numerical analyses reported herein were newly performed for BCUs. Using all of these sources, we first investigated short-term exposures incurred by library workers due to IF-EMF and pulsed EMF. Exposure indices based on mid-term exposure (assuming exposure during employment that may have differed on a weekly basis) were then estimated in order to assess the exposure levels in actual working conditions.

## Materials and methods

### The exposure scenario of library workers

**Figure 1:**
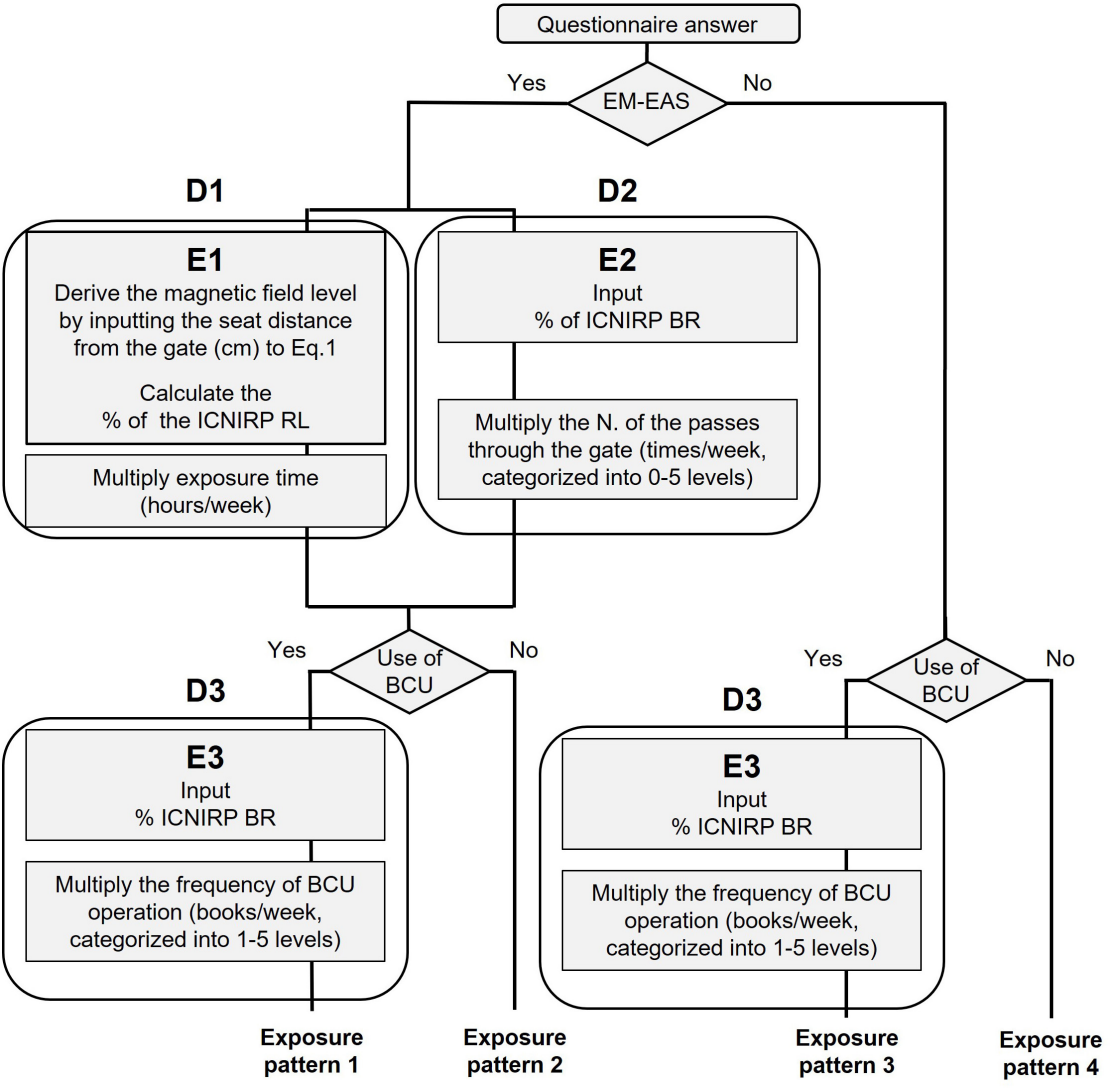
The exposure scenario of library workers.

The occupational exposure scenario considered in this study is shown in Figure 1. The exposure sources of interest in the present study were defined as the EM-EAS gate and BCU. More specifically, we examined IF-EMF exposures from EM-EAS gates and pulsed EMF exposures from BCUs. The present study estimated both short-term exposures (E) and exposure indices based on mid-term exposures (D); these were considered separately as exposure scenario (Figure 1).

The first exposure scenario, short-term exposure (E), was evaluated in terms of ratios in reference to current human protection guidelines (i.e., exposure ratios). More specifically, we referred to both the reference levels (RLs) and basic restrictions (BRs) delineated by the international guidelines recommended by the International Commission on Non-Ionizing Radiation Protection (ICNIRP) (19) in regard to occupational exposures. Three types of short-term exposures were classified as follows: (E1) whole-body exposure from the EAS gate when sitting within 3 m; (E2) local exposure to transient IF-EMF while passing through or beside the EAS gate; and (E3) local exposure on the use of BCUs. The detailed methods for these exposure estimates are described in sections Short-term whole-body exposure from the EAS gate when sitting within 3 m (E1) and Short-term local exposure on passing through or beside the EAS gate (E2).

Previous epidemiologic studies have estimated long-term exposure levels through time integration of short-term exposures (20–22) and examined these exposure estimates as explanatory variables for outcomes of interest. Therefore, exposure indices based on mid-term exposure (D) were also evaluated as a second exposure scenario in order to assess exposure under actual working conditions and to consider the applicability of this exposure assessment methodology to future epidemiological research. Exposure parameters related to work conditions (see section Short-term local exposure on the use of BCUs (E3)) were taken into consideration in exposure estimation.

In this study, we estimated exposures on a weekly basis (termed mid-term exposures) during the course of employment, considering part-time/flexible workers and varying work schedules/allocations. Three types of exposure indices (D1–D3) were identified herein. D1 represents continuous exposure from the EAS gate when sitting within 3 m, while D2 and D3 represent repeated transient exposures due to gate pass (D2) or use of BCUs (D3), respectively. The detailed exposure assessment methods are described in section Short-term local exposure on the use of BCUs (E3).

Four exposure patterns were defined in the present study, according to combinations in use of EAS gates and BCUs (Figure 1). These exposure patterns were defined as follows: exposure pattern 1, combined exposure to IF-EMF and pulsed EMF (E1–E3 and D1–D3); exposure pattern 2, exposure to IF-EMF (E1–E2 and D1–D2) only; exposure pattern 3, exposure to pulsed EMF only (E3 and D3); and exposure pattern 4, exposure to neither IF-EMF nor pulsed EMF. The present study examined potential differences in exposure levels according to exposure patterns.

### Questionnaire distribution

This study was approved by the ethical committee of Shizuoka Graduate University of Public Health (No. SGHIRB#2020005) and was conducted in accordance with the principles of the Declaration of Helsinki. Written informed consent was requested from all participants at the start of the questionnaire.

The invitation letter for partaking in the study was mailed to 4,073 libraries listed as members of the Japan Library Association on the organization's homepage (https://www.jla.or.jp/portals/0/html/jla-e.html). The mailing campaign took place between 2019 and 2020. A link to a web-based questionnaire was distributed to librarians working at all libraries to which the study authors had mailed the institutional questionnaire. Blank responses and one outlier value, described below, were eliminated.

Information on the usage of EAS gates and BCUs was derived from data collected from each library worker's web questionnaire response. In short, the following questions were used in the present study: use of an EAS gate, type of EAS gate, seat distance from the EAS gate (cm), time spent in the worker's seat (hours/week), number of passes through the gate (times/week), use of a BCU, type of BCU, and frequency of BCU operation (number of books/week). We decided to evaluate exposures based on weekly exposure estimates since there were some part-time library employees who worked only a few days a week. The one outlier observed according to this survey (i.e., in regard to time spent in the worker's seat; 87 h/week) was eliminated.

Missing values were estimated using multiple imputation in regard to the number of passes through the EAS gate, since the data distribution showed a non-monotonic pattern (23). Following this, the number of passes through the gate and the frequency of BCU operation, which showed large variability, were divided into six categories for the number of gate passes and into five categories for BCU operation, respectively. Thirty cases in which the EAS gate type could not be identified were classified as “other EAS gates.”

### Assessment of short-term exposures and exposure indices based on mid-term exposures

#### Short-term whole-body exposure from the EAS gate when sitting within 3 m (E1)

In the current study, the lower boundary frequency of IF-EMF was extended from 300 to 220 Hz, the frequency used by a commonly employed EAS gate in Japan. It should be noted that the pulsed EMF originating from BCUs also contains significant frequency components below 300 Hz. The estimated EMF frequencies originating from EAS gates were obtained in this study according to information provided by the manufacturer as well as according to measured data.

The employed measurement method and exposure assessment results were described in the abovementioned study (18). Firstly, the magnetic fields of the gates were measured in accordance with IEC 62369-1 (24), which provides a recommended measurement method for evaluating exposure from short-range devices (including EAS gates). The 45-point average of the magnetic field (*B*_*IEC*_) provides an index of the magnitude of the magnetic field originating from the EAS gate according to the procedure delineated by the IEC 62369-1 (24). The characteristics of distance decay as a function of the distance to the gate were generalized based on the normalized three-dimensional magnetic field distribution of the measured area, That is, the exposure level at the seated position for each respondent was estimated using Equation (1) (18), as follows:


(1) $$\begin{array}{r} \frac{B\left(r\right)}{B_{IEC}}=7.84{\times 10^{4}r}^{-2.89} \end{array}$$

where B(r) represents the estimated magnetic field level at the seated position (μT), B_IEC_ represents the respective results of a 45-point measurement of the target gate conducted based on IEC 62369-1 (24) for gates I, II, IV, V, and VIII (μT), and r represents the seat distance from the EAS gate in cm obtained from the questionnaire survey (see Questionnaire distribution); this was only relevant in cases in which the criterion of r >50 cm was applicable.

For EAS gates yielding a small number of reports (i.e., those with few questionnaire responses) and those that could not be classified for various reasons, the values of representative models (gates I and IV) were substituted based on the similarity of coil diameters and frequencies used in the respective gates. The value for the gate I category was substituted for the gate VIII category (i.e., other gates) because gate I is the most frequently used type of gate in libraries in Japan. Finally, E1 was derived according the relative value of B(r) in comparison to the RL delineated in the ICNIRP 2010 guidelines for occupational exposure (19). Exposure assessment for the general public delineated in this report was not considered, since this study was targeted to library staff. We note that this study separately evaluated E1–E3 exposures. Simultaneous exposures from multiple sources can be evaluated by summing exposure ratios for E1–E3.

#### Short-term local exposure on passing through or beside the EAS gate (E2)

To assess E2, numerical calculations using the impedance method and incorporating complex variables were implemented herein (18). Briefly, to estimate the exposure upon passing through or beside an EAS gate, the induced electric fields in a human body model inside gate II were calculated. The result of the calculation showed that the 99th percentile of the internal electric field in the body was 0.88 V/m, at a frequency of 14 kHz (18). The induced electric field was proportional to the magnitude and frequency of the magnetic field. If the magnetic field distribution was the same, the proportionality coefficient was considered to be the same. Assuming that spatial distributions of magnetic fields from EAS gates are similar near the gate, the induced electric fields in tissue would be proportional to the frequency and magnitude represented by the average IEC value. Here, we assumed that the coil shapes of the EAS gates were similar and that the difference in the coefficients were small. Consequently, the internal electric fields in all other gates were estimated by Equation (2), as follows:


(2) $$\begin{array}{r} E_{\left(target\right)}=E_{\left(ref\right)}\times \left(\frac{f_{\left(target\right)}}{f_{\left(ref\right)}}\right)\times \left(\frac{B_{IEC\left(target\right)}}{B_{IEC\left(ref\right)}}\right) \end{array}$$

where E_(target)_ represents the internal electric field on near-field exposure to the EAS gate (V/m), E_(ref)_ represents the internal electric field of the reference gate (Gate II, 0.88 (V/m)), f_(target)_ represents the operating frequency of the target gate (Hz), f_(ref)_ represents the operating frequency of the reference gate (Gate II, 14 k (Hz)), B_IEC(target)_ represents the IEC value of the target gate (μT), and B_IEC(ref)_ represents the IEC value of the reference gate (gate II, 111 (μT)). The value derived for gate I was substituted for gate VIII (i.e., other gates) because gate I is the most frequently used type of gate in libraries in Japan.

Consequently, E2 was derived according to the exposure ratio in regard to basic restriction (BR) guidelines for occupational exposure delineated in the 2010 ICNIRP guidelines (19).

#### Short-term local exposure on the use of BCUs (E3)

In general, EM-BCUs magnetize the magnetic strips that are attached to books when each book is checked out and demagnetize these strips when the book is returned. Magnetization/demagnetization is performed using a strong pulsed magnetic field (MF). Although the magnetic field in the area where the book is placed is very strong, the exposure to magnetic fields by the human body is quite small because the size of the source is small. In addition to the pulsed MF generated during the processing of strips, the BCU also generates a verification signal (hereinafter referred to as a “detection signal”) to confirm the presence of active strips.

Firstly, the present study evaluated exposures from the pulsed MF during magnetization. This pulse was monophasic. The bandwidth of the magnetizing pulse was broad, with most frequency components below 2 kHz. While the spectrum peak was not entirely clear, we found that it was ~200 Hz.

We note that the waveform of the magnetizing pulse is characterized by its peak value and full width half maximum (FWHM), as this is a monophasic pulse. We selected the BCU I, V, and VIII models as the representative models for this evaluation. The peak values of the magnetizing pulses were measured at 45 points on the measurement grid defined by IEC 62369-1 (24). The spatial average over those 45 points was obtained in the present study.

For BCU I and V, the peak values of the induced electric field in the human body were calculated numerically with the use of an anatomical voxel human model (25). The numerical method was the same as the method described in our previous paper (18), except that those calculations were performed for a number of frequency components of the pulsed waveform in order to reproduce the waveform of the induced electric field in human tissues. The 99th percentile value of the peak induced electric field was obtained and the exposure ratio in reference to the BR delineated by the 2010 ICNIRP guidelines (19) was calculated. Since the BR in the guideline was given by the internal electric field (E_BR_) in terms of the rms values of sinusoidal waves, the E_BR_ values were multiplied by $\sqrt{2}$ to obtain the peak values for BR (i.e., E_BRforpulse_) to compare with the calculated results for the peak internal electric field.

The temporal peak value of B (μT), the FWHM (ms) of the pulse waveform, |dB/ dt|_peak_ (T/s), B${}_{\text{IEC}}\times \sqrt{2}$ (μ T), the calculated peak internal electric field based on the 99th percentile value ((E_in_)_peak_ (V/m)), the internal electric field corresponding to BR of occupational exposures according to the abovementioned ICNIRP guidelines (E_BRforpulse_ (V/m)), E3, and the exposure ratio (%) in reference to the BR delineated in the ICNIRP guidelines are summarized in Table 1.

**Table 1:** Exposure assessment of the magnetic pulse emitted in magnetizing the strip.

**BCU model**	**BCU I**	**BCU V**	**BCU VIII**
Time peak value of B (μT)*^a^	406	338	60
FWHM (ms)	1.95	2.45	0.9
|dB/ dt|_peak_ (T/s)	0.31	0.81	0.16
B_IEC_ X 1/√2 (μT)	287	239	42
(E_in_)_peak_ (V/m)*^b^	0.038	0.054	0.015
E_BR for pulse_ (V/m)*^c^	0.8 X √2	0.8 X √2	0.8 X √2
(E_in_)_peak_/E_BR_ (%)	3.4	4.8	1.3*^d^

^*^^a^ Time peak values at 45 measured points were spatially averaged according to the guidelines set forth within IEC 62369-1.

^*^^b^ The internal electric field calculated according to the peak value. The calculated 99^th^ percentile value of the induced internal electric field in a human body standing in front of a BCU.

^*^^c^ The internal peak electric field corresponding to basic restriction of occupational exposure under ICNIRP guidelines.

^*^^d^ The estimation method is described in section Short-term local exposure on the use of BCUs (E3) of the main manuscript. ICNIRP, International Commission on Non-Ionizing Radiation Protection; IEC, International Electrotechnical Commission; BCU, book-check unit; FWHM, full width at half maximum.

The induced electric field was not calculated for BCU VIII due to a lack of information on the detailed magnetic field distribution. A rough estimate was obtained in this paper, as follows. Specifically, the time derivative of the incident magnetic field was taken as the source of the induced electric field based on the Faraday's law of induction. Therefore, it was expected that the peak induced electric field was proportional to the peak absolute value of dB/dt. The proportional constants for BCU I and V were 0.12 and 0.07 (Vs/Tm), respectively. Thus, a rough estimate for BCU VIII was obtained as 0.015, according to the average of those constants (0.095). Missing data was substituted in this manner. The exposure ratio was calculated for BCU VIII according to a rough estimate of the induced electric field. The value of BCU I was substituted for other all BCUs (i.e., BCU II, III, IV, VI, VII and IX) because, as mentioned above, gate I is the most frequently used type of EAS gate in libraries in Japan.

We next evaluated demagnetization pulses. A demagnetization pulse was defined as an alternating pulse sequence with a damped oscillation waveform. The spectrum was shown to be a rather narrow band, with a peak frequency of ~200 Hz. The peak value and width of the largest pulse in the pulse sequence were almost the same as the respective values of the magnetizing pulses since the same device (i.e., a coil for magnetization/demagnetization) was used. Therefore, the exposures due to pulsed MF for demagnetization were considered the same as those for magnetization with regard to exposure ratios.

Finally, we evaluated the detection signal. The waveforms for the detection signal were different for different BCUs, with 50 Hz continuous waves (CW) for BCU I, 50 Hz burst signals (duty ratio, 50%) for BCU II, and 713 Hz CW for BCU III. Some BCU models allow the magnetic field of their detection signals to be selected as on or off. The exposure ratios of the detected signals were sufficiently small as compared to those of the pulsed MF on processing (9, 10). Therefore, we focused only on magnetization/demagnetization signals in the present study.

#### Exposure indices based on mid-term exposure (D1–D3)

The basic concept underlying the employed exposure indices based on mid-term exposures (D1–D3) was to derive these indices by multiplying short-term exposures (E1–E3) according to weekly basis parameters, such as the duration and frequency of exposure, as derived from the questionnaire responses. For D1, the working condition “time spent in the worker's seat (hours/week)” was used for calculation. Therefore, D1 was represented as % ICNIRP RL × hour/week. E1 values, derived using Eq. 1 (see section Short-term whole-body exposure from the EAS gate when sitting within 3 m (E1)) were multiplied by “time spent in the worker's seat (hours/week)” (Figure 1).

The working condition “the number of passes through the gate (times/week)” was implemented in estimating D2 (Figure 1). D2 was calculated by multiplying the E2 value obtained in section Short-term local exposure on passing through or beside the EAS gate (E2) by this parameter, which was categorized into six groups based on questionnaire responses. Therefore, D2 was represented as % ICNIRP BR × gate pass. The present study did not consider the temporal factor “time required to pass the gate.” D2 was calculated on the assumption that the temporal factor was negligible.

The “frequency of BCU operation (number of books/week)” was considered as the working condition in deriving D3 (Figure 1). D3 was derived by multiplying the E3 values obtained in section Short-term local exposure on passing through or beside the EAS gate (E2) by this parameter, which was categorized into five groups based on questionnaire responses. D3 was represented using the % ICNIRP BR × BCU operation. The present study did not consider the temporal factor (i.e., time required to operate the BCU). D3 was calculated on the assumption that the temporal factor was negligible.

### Statistical analysis

Differences in exposure levels between exposure patterns were examined using the Mann-Whitney *U*-test. IBM SPSS statistical software (v.25, SPSS, Inc., Armonk, NY, USA) was used for the statistical analyses. Statistical significance was set at two-sided *p*-value of <0.05.

## Results

### Exposure estimation for each type of equipment

Table 2 shows the types, specifications, and results for the measurement and calculation of exposure levels at EAS gates as well as on the use of BCUs. The EAS gate type with the highest percentage of respondents was gate I, which accounted for 38.0% of all respondents. The measured gates, gate I (38.0%), gate II (11.5%), gate IV (15.9%), gate V (8.2%), and gate VII (1.4%), accounted for 75.0% of the total responses; this indicated that the selection of representative models (as shown in section Short-term whole-body exposure from the EAS gate when sitting within 3 m (E1)) was appropriate. The relative values for the RL in regard to ICNIRP-based occupational levels at the representative sitting place (i.e., 2 m from the gate) revealed that the gate II showed the highest exposure level (2.0% ICNIRP RL; RL = 100 μT at 14 kHz, Table 2) (19). For the other gates, the exposure levels were lower than 0.2% of the RL, as per the 2010 ICNIRP guidelines (RL = 819.7 or 1,000 μT at 220 or 366 kHz, respectively) (19).

**Table 2:** Types, specifications, and the results of the measurement (E1) and calculation (E2 and E3) of exposure levels from EAS gates and BCUs.

**Equipment**	**Company**	***N***	**% of**	**Frequency**	**B**_IEC_	**ICNIRP**	**E1 % ICNIRP RL**
**type**		**(data)**	**respondents**	**(Hz)**	**(**μ**T)**	**2010 RL**	**(representative value**
						**(**μ**T) (occp)**	at 2 m from the gate)*^f^
**IEC values of EM-EAS gates**							
Gate I	A	79	38.0	220	87	1000.0	0.16
Gate II	A	24	11.5	14 k	111	100.0	2.0
Gate III	A	1	0.5	220	87*^b^	1000.0	0.16
Gate IV	B	33	15.9	366	87	819.7	0.19
Gate V	B	17	8.2	366	68	819.7	0.15
Gate VI	B	4	1.9	366	87*^c^	819.7	0.19
Gate VII	C	3	1.4	220	106	1000.0	0.19
Gate VIII (other gates)*^a^	–	47	22.6	220	87*^b^	1000.0	0.16
Total		208	100.0				
					**Internal**	**ICNIRP**	**E2 %**
					**electric**	**2010 BR**	**ICNIRP**
					**field (V/m)**	**(occp) (V/m)**	BR*^g^
**Internal electric fields induced in the EM-EAS gate**							
Gate I	A	79	38.0	220	0.011*^d^	0.80	1.4
Gate II	A	24	11.5	14 k	0.88	3.8	23
Gate III	A	1	0.5	220	0.01*^d^	0.80	1.4
Gate IV	B	33	15.9	366	0.018*^d^	0.80	2.3
Gate V	B	17	8.2	366	0.014*^d^	0.80	1.8
Gate VI	B	4	1.9	366	0.018*^d^	0.80	2.3
Gate VII	C	3	1.4	220	0.013*^d^	0.80	1.7
Gate VIII (other gates)*^a^	–	47	22.6	220	0.011*^d^	0.80	1.4
Total		208	100.0				
					**E3 % ICNIRP**		
					BR*^g^		
**Internal electric fields induced in use of BCU**							
BCU I	A	123	41.6	–	3.4		
BCU II	A	2	0.7	–	3.4*^e^		
BCU III	A	5	1.7	–	3.4*^e^		
BCU IV	A	8	2.7	–	3.4*^e^		
BCU V	A	80	27.0	–	4.8		
BCU VI	B	7	2.4	–	3.4*^e^		
BCU VII	B	1	0.3	–	3.4*^e^		
BCU VIII	C	32	10.8	–	1.3		
BCU IX (other BCUs)	–	38	12.8	–	3.4*^e^		
Total		296	100.0				

^*^^a^“Other gates” include 30 cases in which the type of EAS gate could not be identified.

^*^^b^The value of gate I was substituted.

^*^^c^The value of gate IV was substituted.

^*^^d^Values were derived from Equation (2) in section Short-term local exposure on passing through or beside the EAS gate (E2).

^*^^e^The value of BCU I was substituted.

^*^^f^Relative values in reference to ICNIRP-derived occupational levels in regard to the RL at a representative sitting place (i.e., 2 m from the gate).

^*^^g^Relative values in reference to ICNIRP occupational levels (BR). BCU, book-check unit; EAS, electronic article surveillance gates; IEC, International Electrotechnical Commission; ICNIRP BR, basic restriction of International Commission on Non-Ionizing Radiation Protection (19); INCNIRP RL, reference level of International Commission on Non-Ionizing Radiation Protection (19).

The same tendency was observed for exposure levels on passing through or beside the EAS gate. The highest exposure level was found at gate II. However, this value was still lower than the permitted level (i.e., 23% of the 2010 ICNIRP BR) (19). For the other gates, the exposure levels were found to be 1.4–2.3% of the BR as delineated in the 2010 ICNIRP guidelines (19).

The BCU type with the highest percentage of respondents was BCU I, which accounted for 41.4% of all respondents. The first three answers excluding BCU VIII (i.e., other BCUs) were BCU I (41.4%), BCU V (27.1%), and BCU VIII (10.8%); these BCU categories covered 79.3% of the total responses. This result indicates that the selection of representative models in section Short-term local exposure on passing through or beside the EAS gate (E2) was appropriate. The highest exposure level was found for BCU V. However, this value was still considerably lower than the permitted level (4.8% of the 2010 ICNIRP BR) (19). For the other BCUs, the exposure levels were found to be 1.5 or 3.4% of the BR, as delineated in the 2010 ICNIRP guidelines (19).

### Questionnaire findings

There were a total of 548 valid questionnaire responses. Table 3 characterizes exposures as reported in the questionnaire. The details of answers in regard to exposure originating from the EAS gate when sitting within 3 m of the gate, the type of gate, and the use and type of BCU are presented in Table 3. Exposure patterns were defined according to the combination of these answers. A total of 238 of the 548 responses (43.4%) indicated exposure originating from any type of EAS gate when sitting within 3 m of the gate. Of these 238 cases, EM-EAS gates were used in 178 cases (74.9%), indicating a high prevalence of IF-EMF exposure among library workers in the present study. Respondents who used EM-BCUs (a source of pulsed magnetic fields) comprised 295 of 548 cases (53.8%), indicating that consideration of pulsed EMF exposure is necessary as well (i.e., apart from IF-EMF exposure).

**Table 3:** Characterization of the exposure.

Total no. (data)	548							
**Exposure from the EAS gate due to sitting within 3 m**		**Gate type**		**Use of BCU**				**Exposure pattern**
Yes	238	EAS	178	Yes	161	EM	153	1
						RF	3	2
						Other	5	2
				No	17	None	17	2
		RFID	22	Yes	17	EM	2	3
						RF	13	4
						Other	2	4
				No	5	None	5	4
		Other cardreader, flapper	8	Yes	7	EM	7	3
						RF	0	4
						Other	0	4
				No	1	None	1	4
		Don't remember/other	30	Yes	27	EM	12	1
						RF	15	2
						Other	0	2
				No	3	None	3	2
No	310			Yes	161	EM	121	3
						RF	22	4
						Other	18	4
				No	149	None	149	4

The details of answers (exposure from an EAS gate due to sitting within 3 m of the gate, the type of gate, and the use and type of BCU) are presented here. Exposure patterns were defined according to the combination of these answers. BCU, book-check unit; EAS, electronic article surveillance; EM, electromagnetic-type; RF, radio frequency-type; RFID, radio frequency identifier.

With respect to the distribution of each exposure category among the study respondents, ~61.7% of the respondents were found to have IF-EMF and/or pulsed EMF exposure. Of these, 30.1% of all respondents were categorized into exposure pattern 1, which represented combined exposure to IF-EMF and pulsed EMF. Exposure pattern 2, that is respondents with only IF-EMF exposure, accounted for 7.8% of the responses. Exposure pattern 3, wherein respondents were exposed to pulsed EMF only, accounted for 23.7% of the responses. Exposure pattern 4 accounted for 38.3% of the responses. Please note that the total does not reach 100% due to rounding.

When evaluating working conditions, “seat distance from the EAS gate (cm)” showed the following distribution: 0–50 (1.0%), 51–100 (10.6%), 101–150 (8.7%), 151–200 (33.7%), 201–250 (9.6%), and 250–300 (36.5%). Opportunities to operate in the vicinity of the gate (<50 cm) were rare. Moreover, “time spent in the worker's seat (hours/week)” was distributed as follows: 0–10 (61.1%), >10–20 (20.2%), >20–30 (5.8%), >30–40 (9.1%), >40–50 (3.4%), and >50–60 (0.5%). Exposure from the EAS gate when sitting within 3 m occurred mostly at a relatively far distance (100–300 cm) and the exposure time was also moderate (<40 h/week). In two cases, many hours of exposure were detected in seating areas located within 50 cm from the gate (i.e., 35 and 42 h per week of exposure under these conditions).

The frequency of passing through the gate (times/week) was distributed as follows: 0 (2.4%), 1–10 (16.9%), 11–20 (15.0%), 20–25 (25.6%), 25–30 (18.4%), and >30 (21.7%). A total of 97.6% of the respondents passed through the gate at least once a day, indicating the necessity for exposure assessment of D2 in addition to that of D1.

The distribution of the frequency of BCU operation (books/week) was 0–20 (21.4%), 21–50 (21.4%), 51–100 (19.7%), 101–299 (18.6%), and >300 (19.0%). These results showed a wide variation in values among respondents.

### Short-term exposures (E1–E3) and mid-term exposures (D1–D3) among library workers

Table 4 shows a summary of the estimated short-term exposures (E1–E3) among library workers. The median relative values for E1–E3 in reference to the 2010 ICNIRP guidelines (19) were as follows: exposure pattern 1, lower than 1% of the RL (the actual value was 0.2% for E1, 1% of the BR for E2, and 3% of the BR for E3); exposure pattern 2, lower than 1% of the RL (the actual value was 0.2% for E1, 1% of the BR for E2; exposure pattern 3, 3% of the BR for E3). None of the cases exceeded the levels permitted by the ICNIRP guidelines. The highest contribution toward exposure was found in E3, whereas exposure from EM-EAS gates did not contribute as much as exposure from BCUs (see Table 4). Regarding IF-EMF exposure, we found that the exposure levels were higher in E2 than in E1, and this trend was common between exposure patterns 1 and 2. Statistical significance was observed only in E2 (pattern 1 vs. pattern 2, *p* <0.05 using the Mann-Whitney U–test).

**Table 4:** Summary of short-term exposures (E1-E3) among library workers.

**Exposure pattern**	**Total**	**E1**	**E2**	**E3**
**Relative value to ICNIRP RL (%) for E1 and BR (%) for E2 and E3**				
Total (*n* = 338)	Median	0	1	3
	Average	1	4	4
	SD	1	7	1
	Max	15	23	5
	Min	0	1	1
Exposure pattern 1 (*n* = 165)	Median	0	1	3
	Average	1	4	4
	SD	2	7	1
	Max	15	23	5
	Min	0	1	1
Exposure pattern 2 (*n* = 43)	Median	0	1	–
	Average	0	4	–
	SD	1	6	–
	Max	5	23	–
	Min	0	1	–
Exposure pattern 3 (*n* = 130)	Median	–	–	3
	Average	–	–	3
	SD	–	–	1
	Max	–	–	5
	Min	–	–	1

ICNIRP BR, basic restriction of International Commission on Non-Ionizing Radiation Protection (19); INCNIRP RL, reference level of International Commission on Non-Ionizing Radiation Protection (19); SD, standard deviation.

When evaluating distributions of E1–E2 values, 99.0% of respondents had an E1 exposure level below 10% RL, as per the 2020 ICNIRP guidelines (19). The rest had an E1 exposure level between 10 and 20% of the RL, as per these same guidelines. For E2, 88.5% of the respondents showed E2 exposure levels below 10% of the BR specified in the relevant guidelines. The value for the rest of the respondents (11.5%) was 22% of the BR; this represents the value derived for gate II (Table 2). The distribution of E3 values was similarly within 10% of the BR (as per the relevant guidelines), since all BCU exposures were within 1.5–5.0% of the permitted levels (Table 2). These results suggest that short-term exposures from EM-EAS gates and from BCUs were below 10% of the guideline-specified levels in most cases.

**Figure 2:**
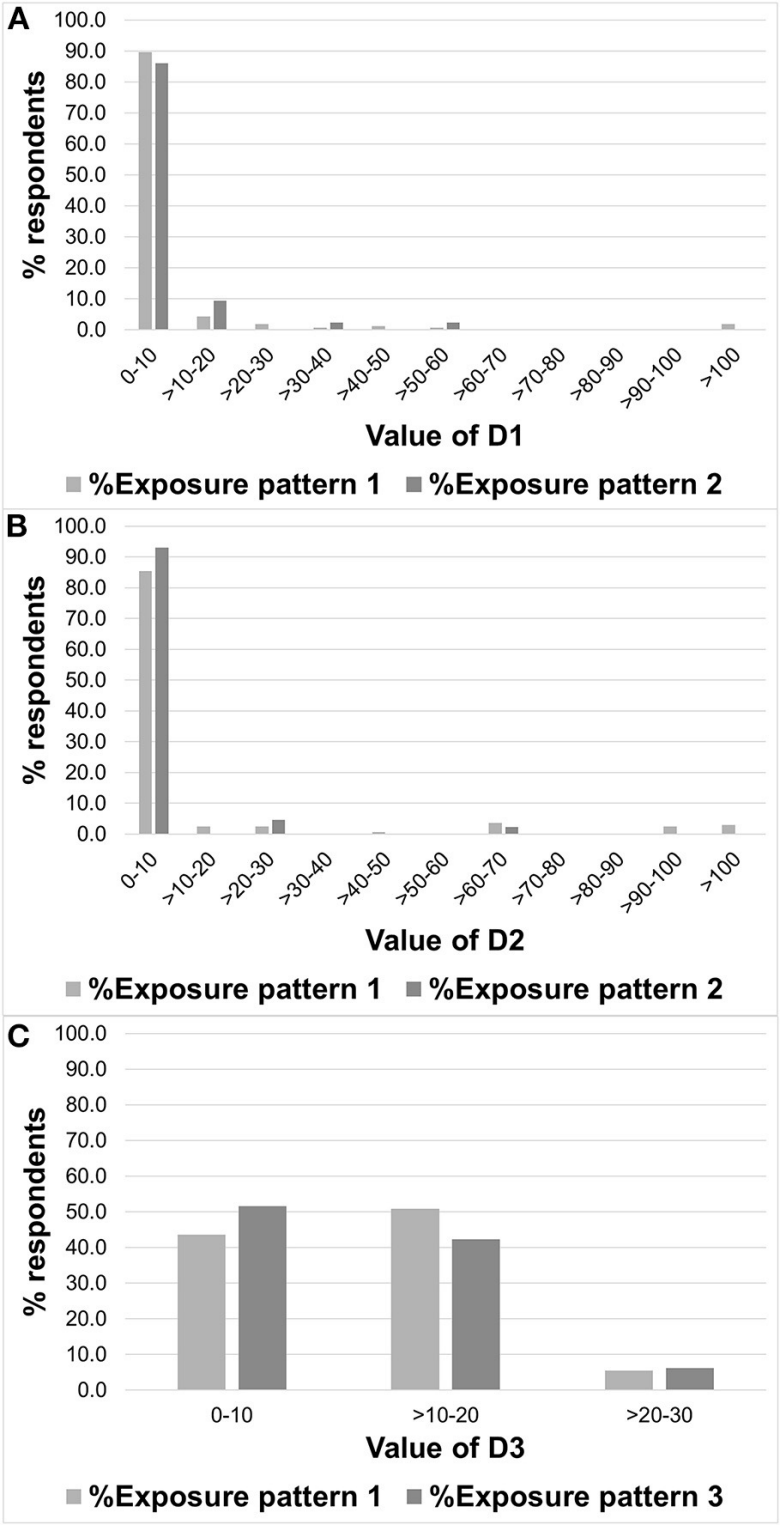
Distribution of D1–3. **(A)** D1, **(B)** D2, and **(C)** D3.

Table 5 and Figure 2 show the values and distributions for D1–D3. The median D1 value was 5, and more than 85% of respondents' values were below 10; in contrast, the maximum value was 513 (Figure 2A). These results indicated that levels of continuous exposure were low in most cases. The same tendency was observed with repetitive short-term exposure from EM-EAS gates, as the median D2 value was 5.4 and more than 85% of respondents' values represented low-level exposures (i.e., below 10); this result was the same as for D1 (Figure 2B). However, as shown in Figures 2A,B, there were several cases wherein D1 and D2 showed very high levels (above 100). In contrast, the median value for D3 was 11, and most of the respondents' D3 values were higher than for D2 (Figure 2C). There was no statistical significance detected for D1 (pattern 1 vs. pattern 2), D2 (pattern 1 vs. pattern 2), or D3 (pattern 1 vs. pattern 3).

**Table 5:** Summary of exposure indices based on mid-term exposures (D1–D3) among library workers.

**Exposure pattern**	**Total**	**D1**	**D2**	**D3**
Total (*n* = 338)	Median	1	5	10
	Average	10	12	11
	SD	51	24	6
	Max	513	116	24
	Min	0	0	1
Exposure pattern 1 (*n* = 165)	Median	1	5	10
	Average	12	13	11
	SD	57	26	6
	Max	513	116	24
	Min	0	0	1
Exposure pattern 2 (*n* = 43)	Median	1	4	–
	Average	4	7	–
	SD	6	11	–
	Max	31	70	–
	Min	0	0	–
Exposure pattern 3 (*n* = 130)	Median	–	–	10
	Average	–	–	10
	SD	–	–	6
	Max	–	–	24
	Min	–	–	1

SD, standard deviation.

## Discussion

The novelty of this study is that we conducted an exposure assessment under the assumption of actual measured working conditions with a focus on combined exposure to IF-EMF and pulsed EMF. We note that the exposure to magnetic fields generated by EAS gates can be divided into two exposure scenarios: exposures in employees passing through the gates and exposures in library staff who work for a long period of time outside of (nearby) these gates.

The former exposure is short-term (26), in contrast to the latter exposure. Several studies have measured or calculated exposures in this situation (6–11). However, we note that this short-term exposure occurs frequently during the workday. Therefore, from a viewpoint of a per working day basis, this exposure is classified as a short-term repetitive exposure. To our best knowledge, the exposure assessment according to this viewpoint has not been conducted to date. The latter situation generally only occurs among employees, and the resulting continuous exposure can be considerable. Regarding this issue, an ICNIRP statement in 2004 pointed out that “for occupational exposures, extended exposure times up to a length of a working shift may occur” (26). This indicates the need for mid- to long-term exposure assessment.

Moreover, to our knowledge, there are currently no studies addressing human exposure to pulsed magnetic fields generated by BCUs. However, library staff routinely perform magnetic tag processing using BCUs. Therefore, processing BCUs should also be considered as an occupational short-term repetitive exposure. In consideration of these situations, the present study focused on short-term repetitive exposure due to gate pass (D2) and the use of BCUs (D3), and continuous exposure from EAS gates when sitting within 3 m of the gate (D1) in order to comprehensively evaluate work-associated exposures. Mid-term exposure duration based on weekly variation was set for D1–D3 in order to consider the diversity of work styles and library business days for library workers.

Previous studies have evaluated long-term exposure by considering actual exposure conditions (e.g., time integration) (19–21). Similar to previous studies, this study assumed D1–D3 exposure estimates using values derived by multiplying E1–E3 exposure parameters by weekly-basis exposure parameters obtained from questionnaire responses, although the exposure duration of the present study was set to 1 week. As a result, continuous exposure (D1) and repeated transient exposure (D2 and 3) were evaluated individually. As shown in Table 5 and Figure 2, the levels of both D1 and D2 were low in most cases of IF-EMF exposure. However, careful consideration of exposure levels is essential for library workers because some workers showed more exposure levels of more than 10 times the respective median value. For example, the values derived for D1 showed a large variation (Table 5). As described in 3.2, close proximity and elevated exposures occurring due to long working hours were present in some of the evaluated workers. These results suggest the need to better characterize strong continuous exposures under certain working conditions.

When evaluating the two short-term and repetitive local exposures (D2 and D3), we found that these exposure parameters were similar in terms of incurred repeated exposures over a short period of time. However, these exposures were evaluated separately in the present study because the time factor (i.e., the time required for gate pass or BCU processing) was not considered. We note that in comparison with D2 and D3, the median value of D3 was ~2 times higher than that of D2 (D2: 5 vs. D3: 10), although the maximum value of D2 was more than four times greater than that of D3 (D2: 116 vs. D3: 24). This was caused by variations in E2 among EAS gates (1.4–23% BR, Table 2), as the number of gate assess (times/week) was distributed equally in this study (as described in 3.1). As shown in Figure 2B, bimodal exposure patterns were observed in D2, and the higher exposure group was considered to have a greater possibility of adverse health effects. Therefore, although IF-EMF exposure from all equipment evaluated in the present study complied with the ICNIRP 2010 guidelines (19), gaining an understanding of the exposure environment is necessary in order to protect employees from excess electromagnetic exposure. Detailed product information provided by manufacturers would aid in gaining understanding in this regard. It may also be necessary to evaluate these two types of exposures in an integrated manner within future research.

Moreover, although D3 appears to be more dominant than D2 for repeated transient exposures (Table 5), it should be noted that the present study assumed that the number of BCUs used by employees was the same as the frequency of BCU operation. In a real working situation, several books are frequently processed simultaneously by the BCU. Therefore, the D3 value will change if we assume the simultaneous processing of books in the operation of the BCU. Additional understanding of working conditions will lead to more accurate exposure assessment. Also, since D1–D3 exposure occur simultaneously in library work, seeking a method for evaluating D1, D2 and D3 simultaneously is necessary within future research.

Essentially, EAS gates and BCUs are used together. Therefore, the communication methods for EAS gates and BCUs are generally the same for a given library (e.g., in the case of the use of an EM-EAS gate and an EM-BCU). However, as shown in Table 3, there were some cases in which there was no correspondence. This may be due to the fact that the questionnaire does not fully reflect the presence of multiple EAS gates or BCUs. When there were multiple EAS gates at a given library, the respondents were asked to provide an answer in regard to the closest EAS gate type; for BCUs, participants were asked to provide an answer regarding the type of BCU they normally used, even if there were multiple BCUs present in the same library.

We note that, in the present study, four exposure patterns were assumed based on combinations of usage of EAS gates and BCUs. Differences in exposure levels depending on exposure patterns were observed only in E2 (pattern 1 vs. pattern 2, *p* < 0.05, Mann-Whitney U-test). This suggests that the factors related to exposure (e.g., exposure opportunities and exposure devices) were evenly distributed in most cases, with the exception of E2.

This study has several limitations. First, we estimated values for gates and BCUs, and actual measurements and calculations were not completed. We also assumed that the human body was located 10 cm from the gate for the purpose of exposure assessment. In addition, the number of gate passes and BCU operations were substituted by estimating missing values, resulting in some uncertainty in exposure estimation. Moreover, the present study did not consider temporal factors (e.g., the time require to pass the gate or operate BCUs) in the assumptions for both D2 and D3, which may have led to some inaccuracy in exposure evaluation. We recommend that this factor be evaluated in future research. In the future, it will also be necessary to verify the accuracy of this exposure estimation methodology by conducting field surveys using personal exposure meters.

However, we emphasize that this is the first study to examine combined exposure to IF and pulsed EMF among library workers in Japan assuming actual working conditions. Considering this strength, the present study fills a significant lacuna in the literature and provides a valuable contribution to the state of knowledge on this topic.

## Conclusion

The present study conducted an assessment of combined exposures to IF-EMF and pulsed EMF among library workers in Japan through the evaluation of both short-term exposures (E1–E3) and exposure indices based on mid-term exposures (D1–D3); assumptions relevant to exposure levels were based on actual working conditions. The results indicated that the short-term exposures from EM-EAS gates (E1 and E2) or BCUs (E3) were below 10% of the limit specified in the relevant guidelines in most cases, and that the ICNIRP limit was not exceeded in any case. When considering exposure indices based on mid-term exposures, continuous exposures (D1) and repeated transient exposures to IF-EMFs (D2) were low in most cases. However, careful attention must be paid to exposure levels in library workers, as some of these workers showed exposure levels of more than 10 times the median value. In addition, we note that the repeated transient exposure from pulsed EMF (D3) was greater than that of IF-EMFs in the present study. Gaining a greater understanding of working conditions will lead to more accurate exposure values. These results provide useful information for future epidemiological studies.

## Data availability statement

The raw data supporting the conclusions of this article will be made available by the authors, without undue reservation.

## Ethics statement

This study was approved by the Ethical Committee of the Shizuoka Graduate University of Public Health (No. SGHIRB#2020005) and was conducted in accordance with the principles of the Declaration of Helsinki. Written informed consent was requested from all participants at the beginning of the questionnaire.

## Author contributions

SY-S, NK, and MT contributed to the study conception and design. MI, KE, AA, and KW measured and numerically calculated exposures from EM-EAS gates and BCUs. All authors contributed to the article and approved the submitted version.

## Funding

This work was supported by the Ministry of Internal Affairs and Communications (JPMI10001), Japan.

## Conflict of interest

The authors declare that the research was conducted in the absence of any commercial or financial relationships that could be construed as a potential conflict of interest.

## Publisher's note

All claims expressed in this article are solely those of the authors and do not necessarily represent those of their affiliated organizations, or those of the publisher, the editors and the reviewers. Any product that may be evaluated in this article, or claim that may be made by its manufacturer, is not guaranteed or endorsed by the publisher.
